# The Effect of taVNS on the Cerebello-Thalamo-Cortical Pathway: a TMS Study

**DOI:** 10.1007/s12311-023-01595-5

**Published:** 2023-08-28

**Authors:** Vesna M. van Midden, Zvezdan Pirtošek, Maja Kojović

**Affiliations:** 1 Department of Neurology, University Medical Centre Ljubljana, Ljubljana, Slovenia; 2 Medical Faculty, University of Ljubljana, Ljubljana, Slovenia

**Keywords:** Vagal nerve stimulation, Transcutaneous electric nerve stimulation, Transcranial magnetic stimulation, Vagus nerve

## Abstract

fMRI studies show activation of cerebellum during transcutaneous auricular vagal nerve stimulation (taVNS); however, there is no evidence whether taVNS induced activation of the cerebellum translates to the cerebellar closed loops involved in motor functions. We assessed the propensity of taVNS at 25 Hz (taVNS25) and 100 Hz (taVNS100) to modulate cerebello-thalamo-cortical pathways using transcranial magnetic stimulation. In our double blind within-subjects study thirty-two participants completed one visit during which cerebellar brain inhibition (CBI) was assessed at baseline (no stimulation) and in a randomized order during taVNS100, taVNS25, and sham taVNS (xVNS). Generalized linear mixed models with gamma distribution were built to assess the effect of taVNS on CBI. The estimated marginal means of linear trends during each taVNS condition were computed and compared in a pairwise fashion with Benjamini-Hochberg correction for multiple comparisons. CBI significantly increased during taVNS100 compared to taVNS25 and xVNS (*p* = 0.0003 and *p* = 0.0465, respectively). The taVNS current intensity and CBI conditioning stimulus intensity had no significant effect on CBI. taVNS has a frequency dependent propensity to modulate the cerebello-thalamo-cortical pathway. The cerebellum participates in closed-loop circuits involved in motor, cognitive, and affective operations and may serve as an entry for modulating effects of taVNS.

## Introduction

Transcutaneous auricular vagal nerve stimulation (taVNS) is a noninvasive electrostimulation technique with a propensity to modulate upstream vagal afferents. The main hypothesis for the behavioral outcomes of noninvasive VNS (nVNS) is the modulation of diffuse neuromodulatory systems, including the noradrenergic, cholinergic, and serotonergic system [1–7], setting the stage for the use of taVNS as a symptomatic treatment in various neuropsychiatric disorders. First neurophysiological evidence that taVNS may affect cerebellum has been provided by fMRI studies on healthy subjects, which showed increased cerebellar BOLD signal compared to sham stimulation [5, 8]. A recent study revealed that 15 min of taVNS at 20 kHz produced a sustained decrease in cerebral blood flow in the posterior cerebellum [9]. The cerebellum participates in numerous closed-loop circuits involved in motor, cognitive, and affective operations and therefore could serve as a non-lesioned entry for neuromodulation of cerebellar and extracerebellar pathways [10, 11]. However, there is still no evidence whether taVNS induced activation of the cerebellum translates to the cerebellar closed loops involved in motor, cognitive, or affective functions.

Transcranial magnetic stimulation (TMS) is a well-established neurophysiological technique for studying the brain excitability and connectivity, and has been successfully used to explore the effects of pharmacologic treatments and invasive and noninvasive brain stimulation interventions [12, 13]. Different TMS paradigms may be used to assess the activity of different neurotransmitter pathways at the level of the motor cortex, while two coils paired-pulse techniques allow for assessment of connectivity between brain structures [14]. Connectivity between cerebellum and the motor cortex may be investigated via a dual-coil TMS paradigm, named cerebellar brain inhibition (CBI) [14, 15]. CBI allows us to explore the activity of the cerebello-thalamo-cortical pathway, by applying a conditioning stimulus (CS) above the cerebellum and the test stimulus (TS) above the motor cortex [16, 17].

In the present study, we assessed the ability of taVNS to modulate cerebello-thalamo-cortical pathway. We compared the effect of taVNS at 100 Hz (taVNS100), 25 Hz (taVNS25), and sham (xVNS) on the CBI in the single session.

## Materials and Methods

### Sample Size Calculation

Since no previous study investigated the effects of VNS on CBI, sample size was calculated based on our preliminary data on 5 participants where we found a standard deviation of difference between means of 0.28. To detect a true difference in the mean, 29 subjects would suffice to achieve 80% power with a Type I error of 0.05. To adjust for error due calculations based on preliminary data and missing data from faulty experiments, we increased our sample by 10% and recruited 32 participants.

### Study Design

**Fig. 1:**
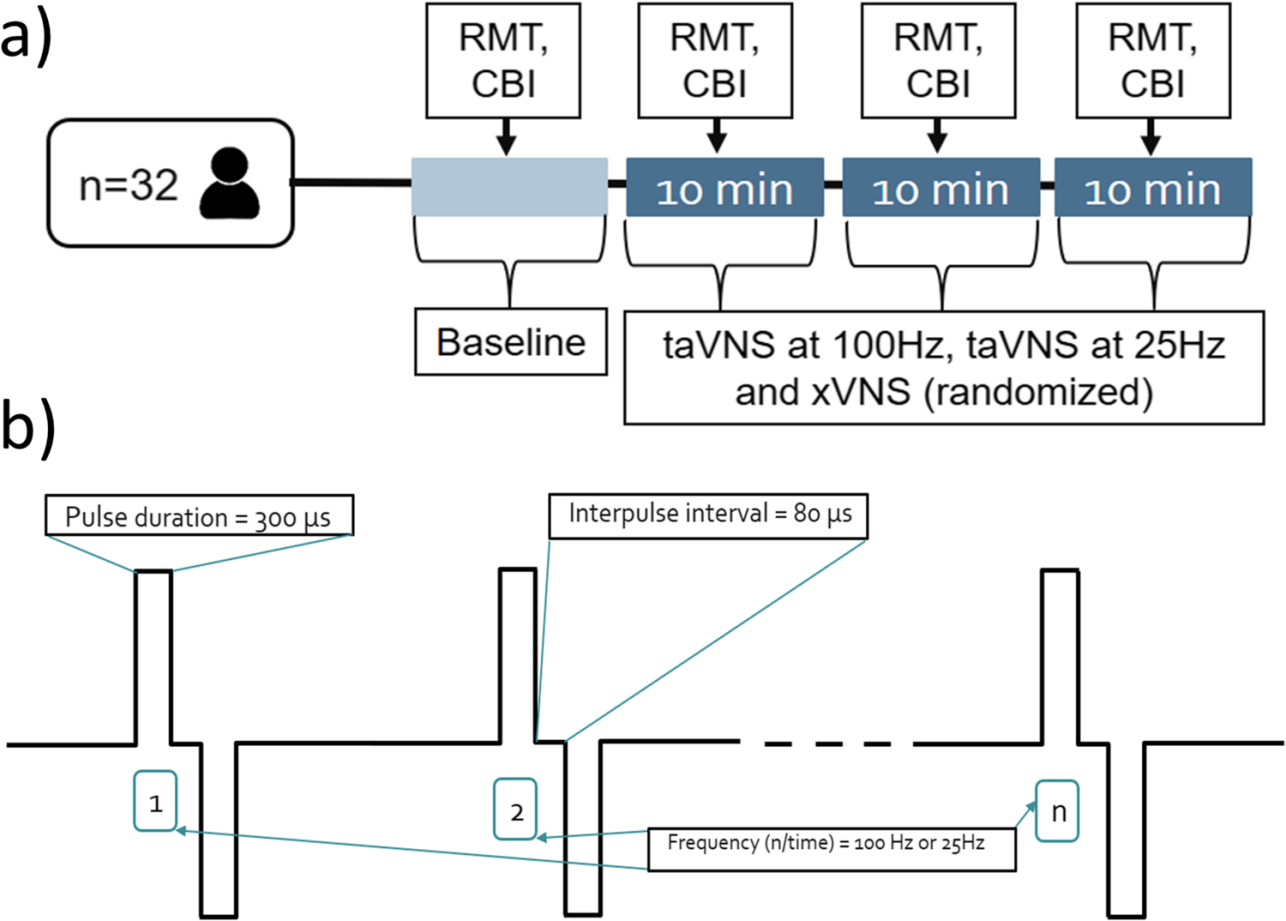
Study setup. **a** Timeline of the experiment, **b** stimulation parameters

In this double blind within subject study, each participant completed one visit during which resting motor threshold (RMT) and CBI were assessed at baseline (no stimulation) and during taVNS100, taVNS25 and xVNS, delivered in a randomized order (Fig. 1a).

### taVNS

We used unilateral, left-sided taVNS, in line with established practice [18]. The electrode was positioned at the left cymba conchae location by the experimenter and the participant was instructed that 3 different stimulation parameters will be compared, including the stimulation below the sensory threshold (which was indeed sham stimulation). To ensure stable contact (in the case of head or neck movements triggered by cerebellar stimulation), the electrode was taped to the ear using micropore and the ear was covered with cotton pads, which were further taped in place with micropore. An EEG cap was placed over the head, covering both ears, adding further stability. During the experiment, the amount of current applied to the electrode was continuously monitored and displayed on a computer screen. This real-time monitoring served as a fail-safe mechanism to ensure that the electrode remained in contact and that the desired level of stimulation was being delivered. During the experiment, the stimulation parameters were wirelessly adjusted by a third person. taVNS was applied through the Nemos® electrode with the following parameters: square-shaped pseudobiphasic pulse, interpulse duration 80 μs, pulse width 300 μs, pulse intensity 0.1 mA above the perceptual threshold (Fig. 1b). Participants received 10 min of each stimulation, during which resting motor threshold was determined and the CBI protocol delivered. For taVNS, stimulation frequencies were either 100 Hz or 25 Hz. For xVNS, first, a few seconds of electrical current were applied, and the participant was asked to confirm that they feel the stimulation, after that the current was turned off.

### Transcranial Magnetic Stimulation and Cerebello-Cortical Inhibition

Previous fMRI studies [5, 8] demonstrated bilateral cerebellar activation during left-sided taVNS. Bashar’s study [8], in particular, revealed that the left-sided taVNS led to more pronounced activation in the ipsilateral cerebellar hemisphere; therefore, we investigated CBI over the left cerebellum. CBI was performed as described by Pinto et al. [19] with CS preceding TS with an interstimulus interval of 5 ms. The TS was applied over the right motor hotspot, using figure 8 shaped coil. The cerebellum was stimulated with the second cone-shaped coil, 3 cm left from the inion. Single TMS pulses over the primary motor cortex (M1) and over the cerebellum were applied using two Magstim 2002 magnetic stimulators with a monophasic current waveform (Magstim Company, Carmarthenshire, Wales, UK). The motor “hot spot” was marked on the participant’s head over the optimal scalp positions for eliciting motor evoked potential (MEPs) of maximal amplitudes in the contralateral (left) first dorsal interosseous (FDI) muscle. Electromyography (EMG) recordings were obtained from the FDI, using AgAgCl surface electrodes. EMG signals were amplified (1000×), bandpass filtered (bandwidth 20 to 2 kHz) with a Digitimer D360 amplifier (Digitimer, UK), digitized at a sampling rate of 5 kHz through a 1401 laboratory interface (Cambridge Electronic Design, Cambridge, UK) and stored on a PC, for “offline” analysis using customized Signal® software version 5.00. The intensity of TS was 120% RMT. RMT were defined as the minimum TMS intensity necessary to evoke a > 50 μV MEP in FDI in at least 3 out 6 trials, and were determined according to the standard procedures [20]. RMT was determined at baseline and reassessed for each stimulation condition. The CS intensity over cerebellum was determined individually, as the highest intensity tolerated by the participant (but at least 5% below brainstem motor threshold) and was kept constant through the experiment. At the beginning of the experiment, any potential cortico-spinal tract (CST) activation was assessed in slightly contracted FDI (sustained muscle activation on EMG) by applying 3 pulses over the cerebellum. If any of the 3 pulses elicited a silent period or a MEP of 50 μV above muscle activity, the CS intensity was lowered for 5% and CST activation reassessed. Fifteen conditioned and 15 unconditioned pulses were delivered. Duration of each TMS measurement was approximately 10 min.

### Statistical Analysis

Statistical analysis was performed in IBM SPSS Package (22; IBM Corp, Armonk, NY). CBI in each condition was expressed as a ratio of mean conditioned MEPs and unconditioned MEPs. The data distribution was assessed with QQ plots, histograms, and Shapiro-Wilk tests.

We first confirmed that participants at a group level manifested baseline CBI (CBI_baseline_), by comparing the mean amplitude of the conditioned MEP with the mean MEP amplitude of the unconditioned test stimulus with Wilcoxon Signed Ranks test. To compare the effect of the three types of taVNS (25 Hz vs. 100 Hz vs. sham) on the CBI values, we built a generalized linear mixed model (GLMM) with a gamma probability distribution, which allowed accounting for the effect of the order of stimulation. Stimulation type, the baseline CBI and the order of stimulation were used as fixed effects, and individual intercepts were added as random effects. We further computed the effect of taVNS on CBI per condition (i.e., the estimated marginal means of linear trends) and compared the differences between slopes in a pairwise fashion with Benjamini-Hochberg correction for multiple comparisons.

Since our GLMM model did not directly compare the CBI values during stimulation to CBI_baseline_ values, we further used paired-samples *t*-tests with the Benjamini-Hochberg correction, to assess the difference between CBI_baseline_ and each of the 3 stimulation conditions.

Finally, we explored whether the current intensity of taVNS or CS intensity had a significant effect on CBI, by building 2 additional separate GLMM models (gamma probability distribution) with current intensity, and CS intensity, respectively, as a fixed effect and individual intercepts as random effects.

To analyze the effect of taVNS on RMT, a generalized linear mixed model (GLMM) with a gamma probability distribution was built with stimulation type, baseline RMT and order of stimulation as fixed effects and individual intercepts as random effects.

## Results

Thirty-one (12 males) participants were included in the final analysis. Data from 1 participant was excluded, because measurements deviated 4 SD from the grand mean. The average participant’ age was 23.6 (ranging from 20 to 31). The mean electrical current intensity for taVNS100 was 540 uA and for taVNS25 505 uA. The mean CS intensity was 71% (SD = 6.733) of maximal stimulator output (MSO).

### CBI

For CBI at baseline, Wilcoxon Signed Ranks Test was significant at *p* = 0.035 (*Z* = −2.108), confirming a suppression of MEP with CS, thus establishing the presence of significant CBI at the baseline.

The GLMM revealed significant main effects of the stimulation type (*p* = 0.000, *F(2, 87)* = 8.596), and the baseline CBI value (*p* = 0.000, *F(2, 87)* = 14.612) on CBI during taVNS and no significant effect of the order of stimulation (*p* = 0.492, *F(2, 87)* = 0.714). The significant effect of stimulation type was due to strongest CBI during taVNS100 (*β* = −0.130, 95% CI [0.249, 0.012], *p* = 0.031), and the weakest during taVNS25 (*β* = 0.112 [−0.006, 0.229], *p* = 0.062), while the coefficient during xVNS was set to 0, because it was redundant. Benjamini-Hochberg corrected pairwise comparisons showed that the CBI value was significantly lower during taVNS100 compared to both taVNS25 and xVNS (*p* = 0.0003 and *p* = 0.0465, respectively) (Table 1). There was no difference in CBI during taVNS25 and xVNS (*p* = 0.065) (Table 1).

**Table 1:** Contrast estimates of pairwise comparisons between estimated marginal means. Data in bold emphasis indicates a significant difference; asterixes reflect the p-value (* corresponds to p < 0.05; ** corresponds to p < 0.01; *** corresponds to p < 0.001).

Pairwise contrasts	Contrast estimate	Std error	*T*	df	Adjusted significance	95% CI	
						Upper	Lower
taVNS100–taVNS25	−0.242	0.058	−4.143	87	0.0003***	−0.358	−0.126
taVNS100–xVNS	−0.130	0.059	−2.194	87	0.0465*	−0.249	−0.012
taVNS25–xVNS	0.112	0.059	1.890	87	0.0650	−0.006	0.229

*taVNS100* - transcutaneous auricular vagal nerve stimulation at 100 Hz; *taVNS25* - transcutaneous auricular vagal nerve stimulation at 25 Hz; *xVNS* - sham stimulation

**Fig. 2:**
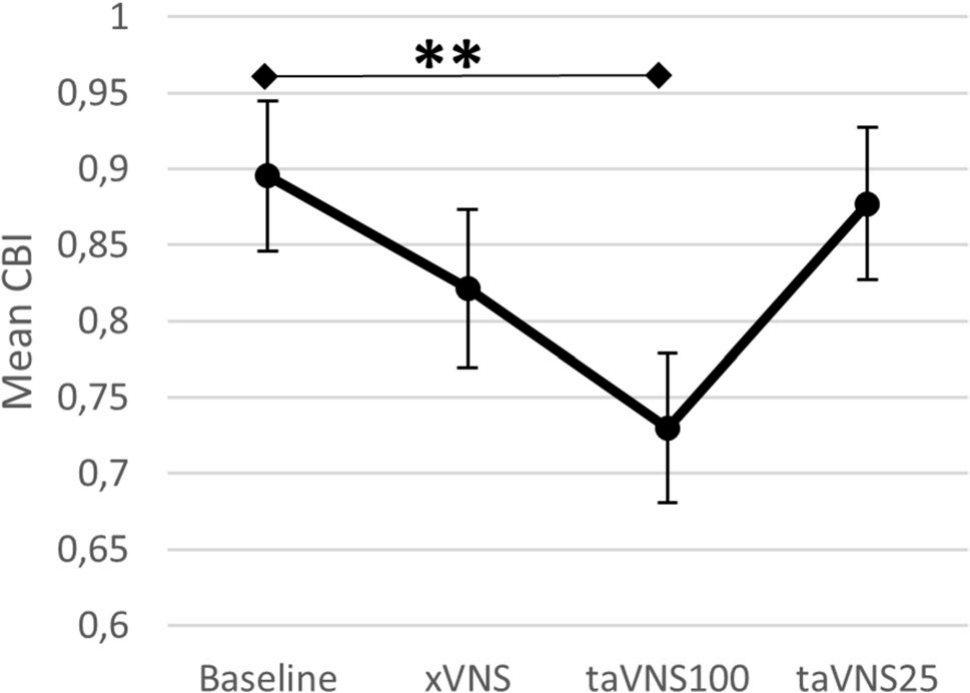
Mean CBI during each condition with standard deviation of the mean. taVNS100, transcutaneous auricular vagal nerve stimulation at 100Hz; taVNS25, transcutaneous auricular vagal nerve stimulation at 25Hz; xVNS, sham stimulation

Comparing CBI during different types of taVNS and CBI_baseline_ (Fig. 2), Benjamini-Hochberg corrected comparisons revealed that CBI_taVNS100_ was stronger compared to CBI_baseline_ (*p* = 0.009, *t*(30) = 3.205), while CBI_taVNS25_ and CBI_xVNS_ were not different from CBI_baseline_ (*p* = 0.752, *t*(30) = 0.3184, and *p* = 0.324, *t*(30) = 1.263, respectively).

Current intensity (*p* = 0.350, *F(2, 90)* = 0.883), or CS intensity (*p* = 0.587, *F(1, 90)* = 0.298) did not significantly affect CBI.

### RMT

We found no significant effect of VNS on RMT (*p* = 0.695, *F(2, 87)* = 0.365). Furthermore, no significant effect of the order of stimulation (*p* = 0.515, *F(2, 87)* = 0.669), current intensity (*p* = 0.168, *F(1, 91)* = 1.930) were found.

## Discussion

Our double blind, sham controlled study provides the first neurophysiological evidence of the effect of taVNS on the cerebello-thalamo-cortical circuit. taVNS100 significantly increased CBI compared to sham stimulation and taVNS25**,** suggesting that taVNS has a frequency-dependent potential to modulate the activity of the cerebello-thalamo-cortical connectivity.

### Possible Mechanism of taVNS Effects on CBI

CBI is a direct measure of the connectivity strength between the cerebellum and M1 [21]. CBI was first described by Ugawa et al., who used transcranial electrical current stimulations as the conditioning stimulus over the cerebellum [21]. Further studies confirmed that TMS is also effective in eliciting CBI [22]. It is widely accepted that the observed inhibition is due to the activation of inhibitory Purkinje cells (PC), which in turn suppress the excitatory input to the ventrolateral thalamus and further to the M1 [22]. Although our study did not address the mechanism of taVNS effect on CBI, several possible explanations may be considered. Within the framework of the prevailing understanding that the effects of noninvasive VNS occurs through activation of the diffuse neuromodulatory systems, taVNS could have activated PCs (and therefore increased CBI) indirectly through noradrenergic locus coeruleus (LC) projections, which strongly innervate PC [23, 24]. Stimulation of LC in animal models results in an enhanced response of PC to climbing fibers, additionally LC activation results in overall inhibition of the activity in the fastigial nucleus [25, 26]. LC activation during taVNS in humans has been repeatedly demonstrated in functional MRI (fMRI) studies [2, 5–7]. Our findings of the selective effect of taVNS at 100 Hz on CBI, with no effect of taVNS at 25 Hz is in line with recent fMRI study which showed that taVNS at 100 Hz lead to the much more robust LC activation compared to 2 Hz, 10 Hz, and 25Hz [6].

Since the auricular branch of the vagal nerve (ABVN) terminates in both the trigeminal sensory nucleus (TGN) and the nuclus tractus solitarii (NTS), we may not exclude that cerebellar modulation, and hence change in CBI with taVNS, occurred through visceral sensory (NTS) or somatosensory (TGN) connection with cerebellum. However, little is known about the function of these connections in humans. TGN forms the trigeminocerebellar tract [27, 28] and NTS forms connections with both the interposed nuclei [29] and the festigial nucleus [30]. Notably, only efferent connections from the cerebellum to the NTS have been described so far.

It is also possible, that modulation of CBI by taVNS occurred at the thalamic level, bypassing the cerebellum, while affecting cerebellar afferents within the ventral intermediate nucleus (VIM). Despite VIM being primarily known for its relay role, recent studies provided evidence that VIM also receives neuromodulatory input from the pedunculopontine nucleus and LC [31]. This mechanism however is less likely, since previous studies consistently showed the activation of the cerebellum with nVNS.

Theoretically, taVNS could have also modulated CBI at the cortical level, by decreasing the motor cortex threshold for evoking CBI. This mechanism seems unlikely, as we found no change in RMT with taVNS in this or our previous study [32]. Importantly, we reassessed RMT within each block of stimulation and accordingly adjusted TS to account for any possible change of cortical threshold for CBI.

### Frequency Specific Effect of taVNS on CBI

The optimal stimulation parameters of taVNS in various disorders are still debatable, and this issue has been addressed in the International Consensus Based Review and Recommendations for Minimum Reporting Standards in Research on Transcutaneous Vagus Nerve Stimulation [18]. In our study we decided to use 100 Hz since this frequency produced the most robust activation of LC in a recent study by Sclocco et al. [6]. We also reasoned, since ABVN is primarily a sensory nerve, stimulations parameters similar to those used in TGN stimulation may be more appropriate [33, 34]. We nevertheless used 25 Hz for comparison, which is currently the most widely used stimulation frequency in taVNS studies, also shown to activate the cerebellum in fMRI studies [5, 8]. We observed a significantly different response to taVNS at 100 Hz compared to 25 Hz, which may be due to frequency dependent activation at the brainstem level or due to specific response of the cerebello-thalamo-cortical pathway to different frequencies. PC are spontaneously active at the rate of 30–150 Hz and respond differently to different climbing fiber and mossy fiber frequencies [22, 35, 36]. In ferrets, frequencies at 0.5–1 Hz produce PC activation, frequencies from 4 to 10 Hz completely silence PC [35]. In humans, direct transcranial alternating current stimulation of the cerebellum at 50 Hz produced a decrease in CBI, whereas 300 Hz produced an increase in CBI [37].

### Possible Clinical Relevance of the Study

Our findings of the propensity of taVNS to modulate CBI may have some relevance for neurological disorders in which dysfunction of the cerebello-cortical system is implicated and in which cerebellum is a potential target for noninvasive stimulation techniques, such as gait disturbances in Parkinson’s disease (PD) [38, 39], essential tremor (ET) [40] or dystonia [41].

### Study Limitations

One limitation of our study is the use of passive instead of active sham, which was done to ensure fixed hotspot electrode location during the whole experiment. Changing of the electrode from cymba conchae to the earlobe for active sham would displace the motor “hot spot” location determined at the beginning of the experiment and marked on a EEG cap. Nevertheless, this should not have affected our findings, since taVNS at 100 Hz was significantly different, not only from sham stimulation, but also from taVNS at 25 Hz.

Very high intensity of TMS pulses over cerebellum may be perceived as unpleasant or painful, which is known drawback of CBI protocol [42]. We used maximal tolerated intensity of TMS over the cerebellum [22] and although on the group level CBI was present, this may have been limiting factor in producing the most effective CBI in all participants. Nevertheless, the average intensity of CS in our study was 70% of MSO, which is comparable with many previous studies [43–46], and we have confirmed that at baseline CBI was present at the group level.

## Conclusion and Summary

Our double blind, sham controlled study showed that taVNS at 100 Hz increased CBI, while taVNS at 25 Hz and sham taVNS did not have effect on CBI. taVNS has a frequency dependent propensity to modulate the activity of the cerebello-thalamo-cortical pathway.

## Data Availability

Data will be made available to researchers upon reasonable request.
